# Wideband MOEMS for the Calibration of Optical Readout Systems

**DOI:** 10.3390/s21217343

**Published:** 2021-11-04

**Authors:** Petr Volkov, Andrey Lukyanov, Alexander Goryunov, Daniil Semikov, Evgeniy Vopilkin, Stanislav Kraev, Andrey Okhapkin, Anatoly Tertyshnik, Ekaterina Arkhipova

**Affiliations:** The Institute for Physics of Microstructures RAS, Academicheskaya Str. 7, 603087 Afonino, Russia; luk@ipmras.ru (A.L.); gorav@mail.ru (A.G.); semikovda@ipmras.ru (D.S.); vopilkin@ipmras.ru (E.V.); kraev@ipmras.ru (S.K.); poa89@ipmras.ru (A.O.); tad1948@mail.ru (A.T.); suroveginaka@ipmras.ru (E.A.)

**Keywords:** optics, fiber-optic sensors, UV-LIGA, MOEMS

## Abstract

The paper proposes a technology based on UV-LIGA process for microoptoelectromechanical systems (MOEMS) manufacturing. We used the original combination of materials and technological steps, in which any of the materials does not enter chemical reactions with each other, while all of them are weakly sensitive to the effects of oxygen plasma. This made it suitable for long-term etching in the oxygen plasma at low discharge power with the complete preservation of the original geometry, including small parts. The micromembranes were formed by thermal evaporation of Al. This simplified the technique compared to the classic UV-LIGA and guaranteed high quality and uniformity of the resulting structure. To demonstrate the complete process, a test MOEMS with electrostatic control was manufactured. On one chip, a set of micromembranes was created with different stiffness from 10 nm/V to 100 nm/V and various working ranges from 100 to 300 nm. All membranes have a flat frequency response without resonant peaks in the frequency range 0–200 kHz. The proposed technology potentially enables the manufacture of wide low-height membranes of complex geometry to create microoptic fiber sensors.

## 1. Introduction

Currently, sensors based on microelectromechanical systems (MEMS) are ubiquitous. The most developed sensors were micro-accelerometers and gyroscopes [1,2,3]. In such systems, reading is carried out in a purely electric manner, most often by changing the capacitance, which is not always convenient and applicable, since it requires the electrical wires for power supply and signal transmission. At the same time, the great potential of such systems has led to the new trend in the sensors development—the development of fiber-optic sensors with a micromechanical sensing element. This enables obtaining a sensor with all the advantages of fiber-optic sensors, such as high sensitivity, the insensibility to the electromagnetic interference, etc., and the advantages of the micromechanical elements, such as small size and adaptive management ability of the sensor parameters by changing its geometry at the engineering steps. The most widespread fiber-optic sensors are micro-accelerometers [4,5,6,7] and pressure sensors [8,9,10]. Previous studies also reported successful implementations of electric voltage sensors [11], chemical sensors [12], acoustic sensors [13].

The characteristic dimensions of sensitive elements in such systems are 10–100 µm. It enables the effective integration of them with an optical fiber and obtain sensors of small sizes. Currently many different methods for the microsensors manufacturing have been developed. The well-known technique is machining by deep reactive-ion etching process [14,15,16]. This is an etch process with high anisotropy used to create deep holes and vertical walls in wafers with high aspect ratios. This technique allows forming of 3D geometry directly in the wafer/substrate. The other one is micromachining [17,18,19,20]. It is a mechanical micro cutting technique (micro milling, micro drilling, etc.) with conventional precision machines. The next one is wet bulk micromachining [21,22] which is based on high anisotropic wet etching and is popular and cost-effective technique for microstructures fabrication.

Currently, the LIGA technology (German acronym for Lithographie, Galvanoformung, Abformung (Lithography, Electroplating, and Molding)) [23,24,25,26,27,28], has acquired great attention. It is based on a sequence of lithography, electroplating and microforming processes that enable the creation of 3D elements with vertical sides and small lateral dimensions. In the process, polymer photoresist, as a rule polymethyl methacrylate (PMMA), exposed by parallel beams of high-energy X-rays. After chemical removal of exposed photoresist, the given 3D structure is coated by metal. Finally, the resist is chemically removed to produce a metallic mold or directly MEMS structure. This technique is used not only for MEMS fabrication but also for many other applications. For example, for THz optics [29], subwavelenght optics [30], micro mass spectrometer [31] and different others.

Currently the modification of the LIGA-UV-LIGA technology [32,33,34,35] has a great popularity. In this technique instead of the X-ray lithography, the ultraviolet (UV) is used. This technology is noticeably cheaper and enables the obtaining of elements of fairly complex geometry.

A modified version of this technology was proposed and implemented in the work. Its enables the obtaining of a free membrane of almost arbitrary geometry. To demonstrate the proposed technology, a matrix of test membranes with electrostatic control and optical readability with flat frequency response in a wide frequency range was created.

## 2. Technology

**Figure 1:**
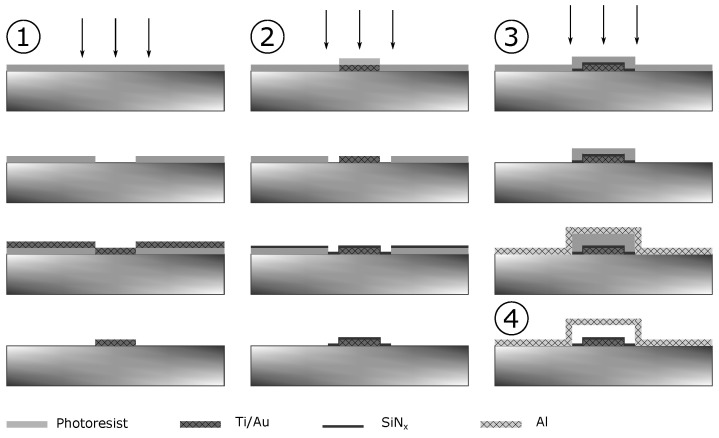
MOEMS forming map. Bottom electrode forming, protective dielectric layer forming, membrane forming, and release of the membrane are indicated by encircled 1, 2, 3, and 4, respectively.

The process worksheet is shown in Figure 1.

The required structure of the microoptoelectromechanical system (MOEMS) was formed in several steps. As a wafer was used an i-GaAs. The manufacturing of an element consists of four main stages:

1. the formation of a bottom electrode;
2. the formation of a layer of the protective dielectric;
3. the formation of the membrane;
4. the release of the membrane.

Stage 1. The bottom electrode was formed by electron-beam deposition of a thin metal layer (in our case, Ti/Au 20/100 nm) and subsequent lift-off process.

Stage 2. To protect against a possible short circuit, which can be caused by a contact of the membrane and lower electrode at large biases, we must apply a layer of protective dielectric. In this case, the dielectric must fulfill two main characteristics: (i) it must be sufficiently thin to maximize the operating range of biases of the membrane, (ii) it must have good dielectric properties and mechanical strength. SiN${}_{x}$ with plasma-enhanced chemical vapor deposition (PE-CVD) was chosen as the dielectric material. Our earlier work on its deposition as a gate dielectric showed excellent dielectric properties of even thin SiN${}_{x}$ layers [36]. At the same time, this material does not chemically react with either the substrate material or the electrode materials and remains stable in the oxygen plasma. The required pattern, as in the first stage, was formed by the lift-off process.

Stage 3. The third stage consists of three steps. In the first step, using photolithography, a sacrificial layer of the photoresist is formed, this layer sets the basic 3D geometry of the future membrane. In the second step, using photolithography, a lateral profile of the membrane is formed. In the third step, an operating layer of metal of the required thickness is deposited; then the final geometry of the membrane is formed using a lift-off process. Please note that in the classical LIGA technology, galvanic deposition with a preliminary spattering of a thin seed layer is used to form an operating metal layer. In our work, thermal evaporation was used to form the operating metal layer. Aluminum was chosen as the material. This approach enables us to obtain a smooth thin homogeneous film with a high response to external voltage and a flat frequency response in the frequency range up to 200 kHz.

**Figure 2:**
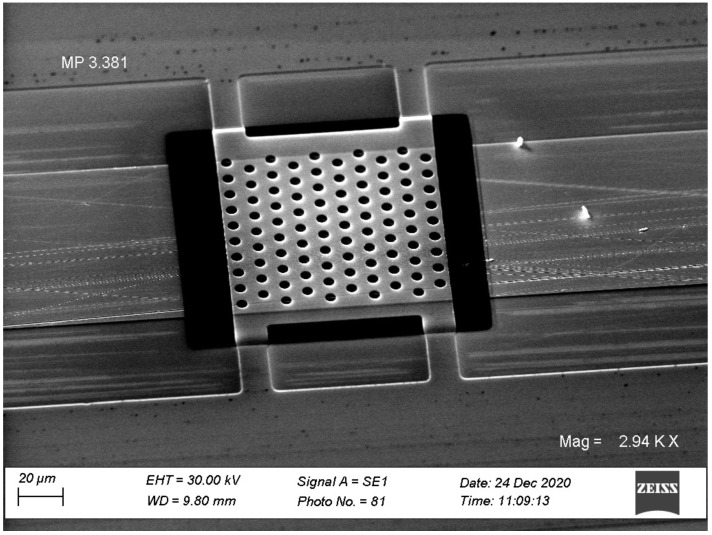
Electronic photograph of the membrane.

Stage 4. The final stage is the removal of the previously formed sacrificial layer of the photoresist, as a result of which the membrane is released from support. As a rule, selective liquid etching was used for this. Here we make the removal of the sacrificial layer of the photoresist by long-time etching in oxygen plasma with low discharge power. Simultaneously aluminum, used as a membrane material, does not react with the plasma and completely maintains its geometry. An electronic photograph of one of the variants of the final element is shown in Figure 2. The size of the membrane is about 100 × 100 µm.

**Figure 3:**
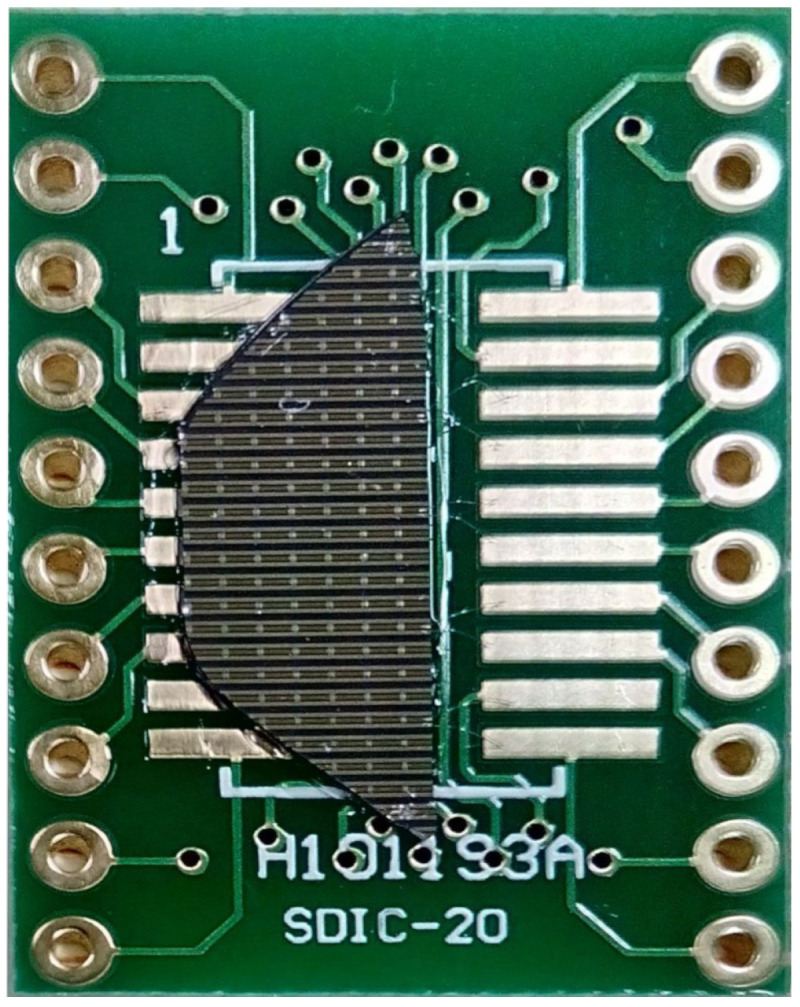
The chip with a set of membranes on the board.

Because the membrane parameters depend on its thickness and support stiffness, which can be adjusted by the width of the skid feet, a set of membranes with different parameters was manufactured on a single chip. Subsequently, the chip was mounted on a printed circuit board, with the possibility of independent excitation of individual rows of membranes (Figure 3).

## 3. Studying Static Parameters

**Figure 4:**
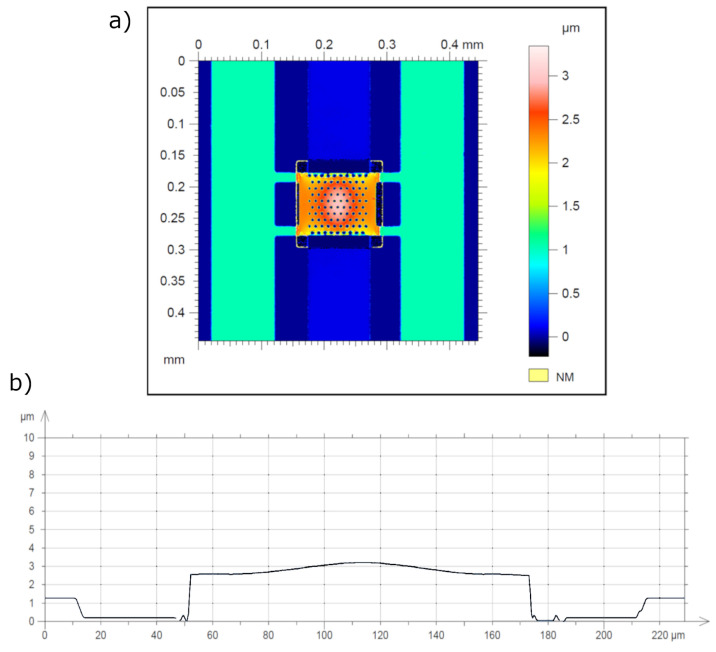
2D membrane profile: (**a**) profile of the membrane surface, obtained on a Talysurf CCI2000 optical profilometer; (**b**) cross-section of the profile in the center from left to right.

Figure 4 shows the profile of the membrane surface, obtained on a Talysurf CCI2000 optical profilometer.

**Figure 5:**
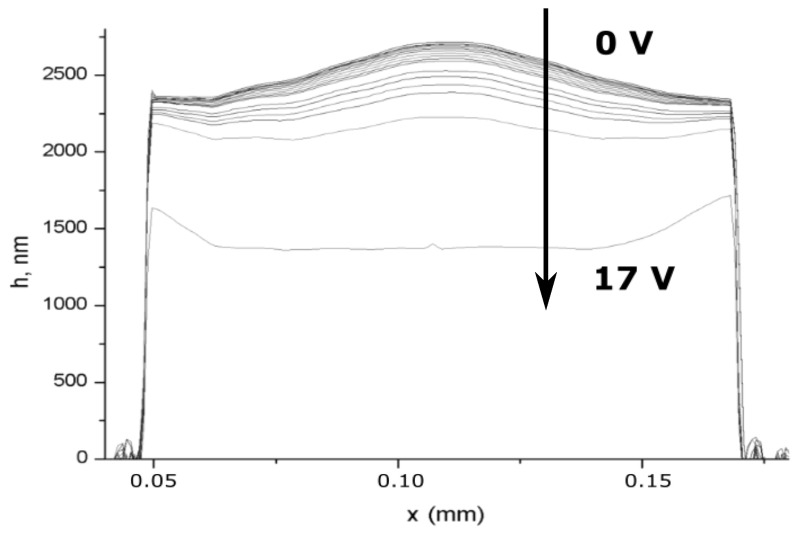
A set of membrane profiles when changing the control voltage from 0 to 17 V.

In general, the membrane surface has a central bulge of about 0.4 μm. Figure 5 shows a set of sequentially taken cross-sections of the profile, when the control voltage is applied.

Because the geometry of the support used by us in the form of lateral feet has a sufficiently high stiffness, when voltage is applied, the outer part bends less than the central one. It allows us using both interference methods for controlling the position of the membrane, when the detection beam is oriented in its central part, and amplitude methods, when using an inclined part of the profile. The bottom profile corresponds to a voltage, applied outside the operating range, at which the membrane falls on the bottom plane.

**Figure 6:**
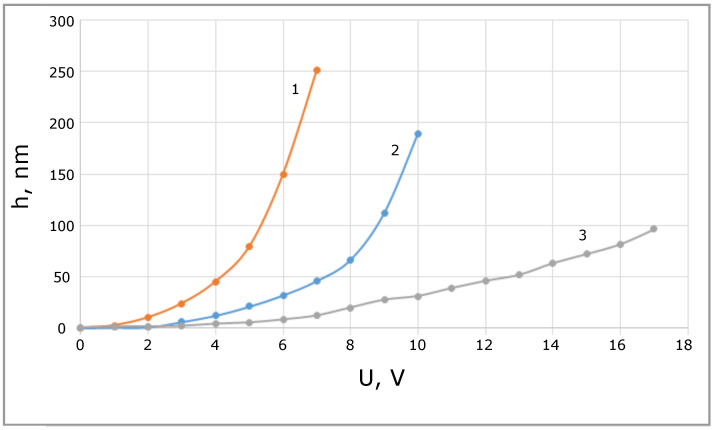
Membrane bias when voltage is applied for different feet widths of 15, 30 and 100 μm are shown by solid lines marked by 1 (orange), 2 (blue), and 3 (grey), respectively.

Figure 6 shows the dependence of the membrane bias on the external stress for a membrane with a thickness of 0.4 μm and different widths of the skid feet of 15, 30 and 100 μm (solid feet).

Increasing the width of the feet increases the stiffness of the membrane and therefore decreases its sensitivity and maximum bias but simultaneously increases the maximum working voltage and the voltage region of the linear response. The use of narrow legs enables sufficiently change the membrane sensitivity by the applied voltage, but it requires operation with low level signals for maintaining linear response. The use of a membrane with solid feet allows maintaining linearity even with sufficiently large signals.

**Figure 7:**
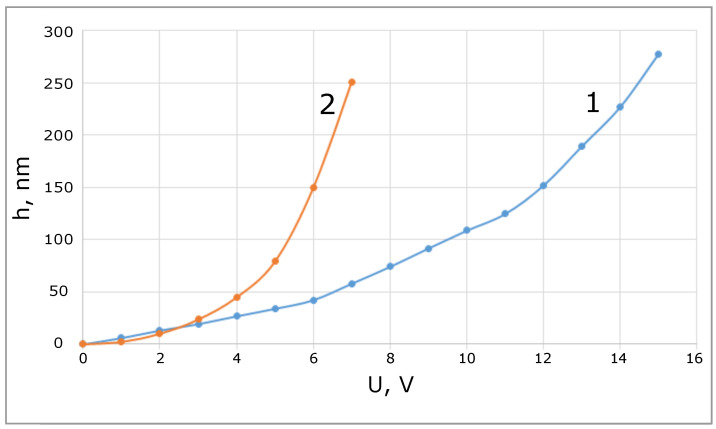
Membrane bias when voltage is applied for two different thicknesses: 1 μm (labeled by 1), 0.4 μm (labeled by 2).

Figure 7 shows a comparison of two membranes with the same geometric parameters, but different membrane thicknesses 0.4 and 1 μm.

In the area of small biases, where the stiffness is determined by the elasticity of the feet, their sensitivity is practically the same. At large biases, a thinner membrane has a significantly greater response with a correspondingly smaller operating range.

**Figure 8:**
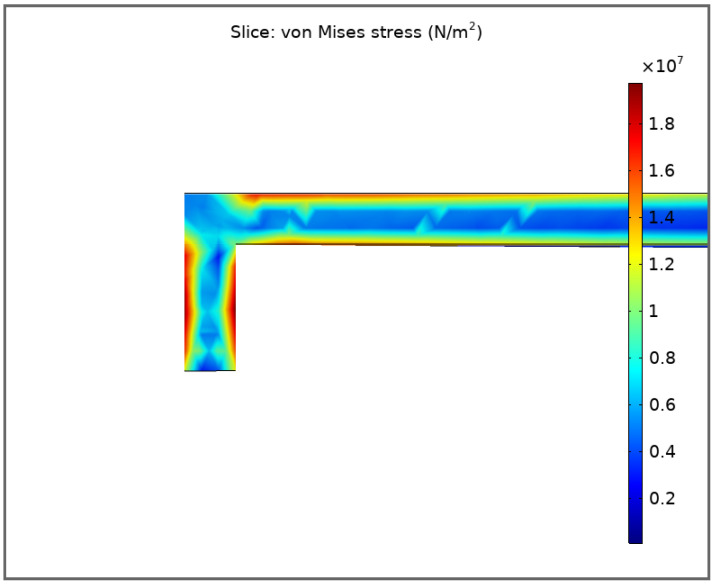
Mechanical stresses at 300 nm deformation.

Figure 8 shows the calculated in COMSOL Multiphysics profile of mechanical stresses for a membrane with a thickness of 0.4 μm at a bias of 300 nm.

The maximum von-Mises stress is about $2\times 10^{7}$ N/m${}^{2}$, which is approximately two times lower than the Al yield point. Thus, in working biases up to 300 nm, the membrane is in the elastic range of stress.

## 4. Dynamic Parameters

**Figure 9:**
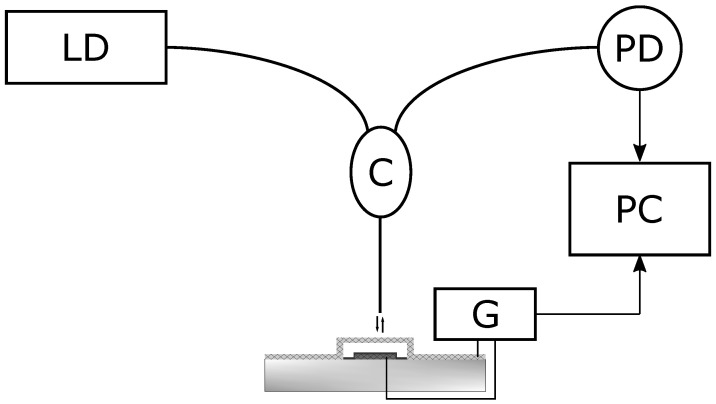
Schematic diagram for frequency response measurement. LD-laser diode, C-optical circulator, PD-photodetector, G-sound generator, PC-personal computer.

To study the frequency response of MOEMS, the following optical circuit was assembled (Figure 9).

**Figure 10:**
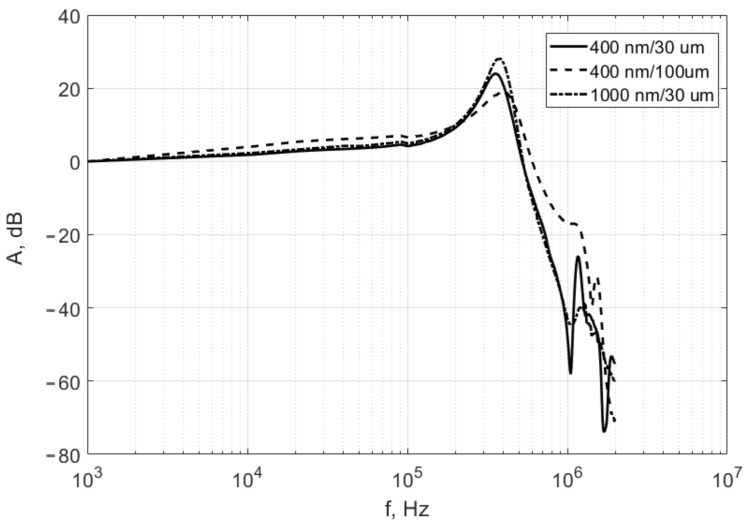
Frequency response of membranes with different parameters. Solid line-thickness is 400 nm, width of skid feet is 30 μm; dashed line-thickness is 400 nm, width of skid feet is 100 μm; dotted line-thickness is 1 μm, width of skid feet is 30 μm.

The light power reflected back from the membrane to the optical fiber depends on the membrane bias due to its reproducible deformation (Figure 5). A sinusoidal signal was applied between the membrane and the lower electrode. We used the Rigol DG822 generator with remote control from the personal computer (PC). The signal had an amplitude of 0.1 V with a bias 2 V. The frequency was scanned in the range from 1 kHz to 2 MHz with a step of 1 kHz. The signal from the photodetector was applied to the analog-digital converter (Adlink PCI-9812A) simultaneously with the signal from generator. An amplitude of the signal was calculated by the digital lock-in algorithm. Figure 10 shows the frequency response of membranes with different parameters.

Curves 1 and 2 correspond to membranes with a thickness of 400 nm and different skid feet widths(solid line-30 μm, dashed line-100 μm); curve 3 corresponds to a membrane with a thickness of 1 μm and feet width of 30 μm. The frequency response varies slightly depending on the membrane parameters. We can see that feet width is the main parameter for the curve shape. Therefore, for membranes of 400 nm/30 μm and 1000 nm/30 μm, the frequency curves are about the same. At the same time, for the membrane 400 nm/100 μm there is a shift of the resonant peak towards higher frequencies due to the greater stiffness of such membrane. Additionally, we can note the smoother curve for the solid feet membrane (100 μm) compared to the membranes with 30 μm feet. Apparently, the additional boundaries of the feet lead to the more complex mode structure of oscillations and, as a result, to the additional resonances. In any case, for all membranes for frequencies up to 200 kHz, the frequency response has a flat character and the total change in the amplitude of the sensitivity does not exceed 8 dB, which is very convenient for the membranes uses as the calibration objects.

## 5. Conclusions

The paper proposes a modification of the UV-LIGA technology, which enables the obtaining of wide membranes of various geometries. An original technological route for the fabrication of the simultaneously low-height and wide structures was developed. This enables the expansion of the range of possible geometries and resulting parameters of the structures you create. At the same time, long-term etching in the oxygen plasma at low discharge power was considered to be the main method of the final removal of the resist, since liquid etching is not very suitable for such geometries.

The main idea was that all materials were completely chemically inert to each other, including the heating process and insensitive to oxygen plasma. It is known that organic materials, including photoresist, can be very efficiently removed in oxygen plasma; however, when removing a resist from wide low-height bridges, its etching rate is quite low (the wider the bridge, the more slowly the resist is removed under the bridge). As a result, membrane materials are forced to remain quite long under the influence of high temperature in a chemically aggressive medium.

The original combination of materials was proposed in the work: GaAs/Au/SiN${}_{x}$/Al. Any pair of materials is inert to each other and insensitive to oxygen plasma. This made it possible to carry out etching for a long time (up to several hours) at low discharge power with the complete preservation of the original geometry, including small parts.

An additional feature of the proposed manufacturing process was the use of thermal deposition of aluminum as a membrane material, instead of electroplating as in classical LIGA. This enables the obtaining of a smooth uniform film in one process, while aluminum, which is quickly coated with Al${}_{2}$O${}_{3}$, is absolutely inert to the oxygen plasma.

As a part of this work, to demonstrate the complete technological cycle, a test MOEMS was manufactured with electrostatic control, optical readability and flat frequency response in the 0–200 kHz band. This MOEMS matrix demonstrates the technological possibility of the proposed route, and it also can be used as a reference object for calibrating optical readout systems in a wide frequency range due to the lack of resonances in the 0–200 kHz band.

The proposed technology potentially will enable the production of solid flat mirrors on an elastic suspension with optics-friendly sizes (tens of microns) and well-developed microsprings and create a fully optical fiber micromechanical accelerometer.

## Data Availability

All evaluated data are presented in this paper in the graphical form. The raw measured data of this study are available on request from the corresponding author.
